# MDS and TP53: When One Hit Just Isn’t Enough…

**DOI:** 10.1097/HS9.0000000000000494

**Published:** 2020-11-06

**Authors:** David G. Kent

**Affiliations:** York Biomedical Research Institute, Department of Biology, University of York, York, UK

In 1971, Alfred Knudson first proposed a “two-mutation hypothesis”—now commonly referred to as the “two-hit hypothesis”—for retinoblastoma, where a tumor suppressor gene gets inactivated in tumors derived from retinal cells. The idea was formulated based on the differential onset of disease in patients who inherited a pre-disposition compared to those that acquired the disease sporadically. The “first hit” turned out to be an inherited loss of function in one allele with the second allele getting mutated later in life. In non-inherited cases, both copies had to be lost in a single cell, making the likelihood of occurrence far lower and the disease substantially delayed in onset. Fast forward to 2020 and we can see catalogues of tumor suppressor genes that follow this pattern, from APC through to TP53 – all with this pattern that requires complete loss of the gene product to have its most damaging, disease accelerating, effects. Concomitant with this list of tumor suppressors is a huge wealth of genomic and clinical characterization that can now let the field determine the true impact of the different mutational states and a recent publication from Bernard et al., highlights the value of such datasets in the context of *TP53* mutations.^1^

TP53 is one of the most studied tumor suppressor genes in the cancer literature, being dubbed the “Guardian of the Genome”,^2^ and strongly associated with sequence specific DNA-binding as well as DNA repair.^3^ As one of the most frequently mutated genes in human cancers, there are numerous types of mutations that have been described,^4^ ranging from heterozygous loss of function, dominant negative missense mutations and complete loss of function with patients commonly classed as in a binary fashion of “p53-mutant” or not. In hematological malignancies, numerous papers have associated p53 mutational status with poor prognosis,^5–7^ but relatively few papers have investigated much beyond the presence/absence of a mutation when it comes to biological and clinical implications in large datasets.

The Bernard manuscript does exactly this in a very large cohort of patients with myelodysplastic syndromes (MDS) by comparing *TP53* allelic status. Comparing >3300 patients with complete clinical and genetic information, the paper divided MDS patients into those with a single hit (one allele of TP53 mutated) versus those with multiple hits (multiple alleles mutated). One third of patients were classed as monoallelic and the other two thirds as multi-hit, the latter of which suggests that the same cell might have lost both copies. The analysis showed that multi-hit TP53 patients were more likely to have complex karyotypes and a significantly higher risk of death and leukemic transformation.

Perhaps more interestingly, the paper also demonstrated that monoallelic *TP53* mutations were virtually indistinguishable from non-mutant *TP53* patients with respect to disease outcome and treatment response. This runs counter to data showing that single copy loss of *TP53* leads to increased propensity to cancer development^8^ and increased accumulation of DNA-damage through reduced nucleotide excision repair,^9^ where one might expect poorer outcomes when a single copy is lost in humans. Also of interest is that a proportion of the *TP53* multi-hit MDS patients might have bi-clonal mutations (ie, 2 different *TP53* mutations in two different cells of origin) meaning that further resolution of the biological and clinical implications might be obtained through newly emerging single cell methods to profile patient samples.^10,11^

Overall, this study is most interesting because it begins to engage with the complexity of single genes and their mutational status rather than binary outcomes of presence/absence. That said, there are further elements of heterogeneity involved (type of mutation, fitness of competing clones, microenvironmental changes, timing with other mutations, etc) that all might influence clinical outcomes. This will require large datasets with carefully curated clinical and genomic information (such as this paper does for MDS) and teams of people to undertake deeper analysis that reaches beyond the top 2 or 3 findings in the first manuscript. Hopefully, this paper stimulates such data mining within its consortium of MDS researchers but also acts to inspire collaborative groups in other hematological malignancies to build sufficiently large, well-characterized datasets with the power to reveal mutational heterogeneity and its potential clinical consequences.
